# Age-dependent plasticity in endocannabinoid modulation of pain processing through postnatal development

**DOI:** 10.1097/j.pain.0000000000001027

**Published:** 2017-08-01

**Authors:** Charlie H-T. Kwok, Ian M. Devonshire, Amer Imraish, Charles M. Greenspon, Stevie Lockwood, Catherine Fielden, Andrew Cooper, Stephen Woodhams, Sarir Sarmad, Catherine A. Ortori, David A. Barrett, David Kendall, Andrew J. Bennett, Victoria Chapman, Gareth J. Hathway

**Affiliations:** a School of Life Sciences, The University of Nottingham, Nottingham, United Kingdom. Kwok now with the Faculty of Veterinary Medicine, University of Calgary, Calgary, AB, Canada; b Centre for Analytical Bioscience, School of Pharmacy, The University of Nottingham, Nottingham, United Kingdom; c Arthritis Research UK Pain Centre, Nottingham, United Kingdom

**Keywords:** Pain, RVM, PAG, Development, Postnatal, Endocannabinoid, Descending control

## Abstract

Significant age- and experience-dependent remodelling of spinal and supraspinal neural networks occur, resulting in altered pain responses in early life. In adults, endogenous opioid peptide and endocannabinoid (ECs) pain control systems exist which modify pain responses, but the role they play in acute responses to pain and postnatal neurodevelopment is unknown. Here, we have studied the changing role of the ECs in the brainstem nuclei essential for the control of nociception from birth to adulthood in both rats and humans. Using in vivo electrophysiology, we show that substantial functional changes occur in the effect of microinjection of ECs receptor agonists and antagonists in the periaqueductal grey (PAG) and rostroventral medulla (RVM), both of which play central roles in the supraspinal control of pain and the maintenance of chronic pain states in adulthood. We show that in immature PAG and RVM, the orphan receptor, GPR55, is able to mediate profound analgesia which is absent in adults. We show that tissue levels of endocannabinoid neurotransmitters, anandamide and 2-arachidonoylglycerol, within the PAG and RVM are developmentally regulated (using mass spectrometry). The expression patterns and levels of ECs enzymes and receptors were assessed using quantitative PCR and immunohistochemistry. In human brainstem, we show age-related alterations in the expression of key enzymes and receptors involved in ECs function using PCR and in situ hybridisation. These data reveal that significant changes on ECs that to this point have been unknown and which shed new light into the complex neurochemical changes that permit normal, mature responses to pain.

## Abstract 2

Supplemental Digital Content is Available in the Text.

Endocannabinoid signalling within brainstem centres that control top-down pain control changes significantly in early life in both rodents and humans.

## 1. Introduction

Pain in infancy is a clinical concern and has been recognised as being suboptimally managed.^58^ Previous studies have shown that pain processing in young mammals is immature: nocifensive withdrawal thresholds are lower, and response magnitudes are greater and longer lasting during early life.^21^ Normal adult processing of noxious sensory inputs requires a constant balance between synaptic excitation and inhibition within the somatosensory pathway.^16,56^ Descending modulatory pathways, specifically the spino-bulbo-spinal loop, plays a key role in modulating spinally mediated nociceptive reflexes.^48^ The periaqueductal grey (PAG) of the midbrain and nuclei within the rostroventral medial medulla (RVM) are pivotal within this loop,^18,32,39,40,43^ as they integrate pain-related activity from forebrain structures and bidirectionally modulate spinal cord dorsal horn (DH) excitability accordingly.^18^ Functional nociceptive processing requires a prolonged period of postnatal maturation, and immature pain behaviours are partly explained by the predominance of synaptic excitation over inhibition within the DH.^1,20,22,28,30,38,39,53^ Opioidergic activity within the descending pathway is one of the major neurotransmitter systems responsible for endogenous pain control,^2,3,43^ and we and others have previously shown that significant postnatal refinement occurs in the opioidergic signalling system.^30,39^

Alongside the opioidergic pain modulatory system, a parallel endocannabinoid (EC) signalling system exists.^12,42,60^ In adult rodents, the administration of synthetic cannabinoid agonists into the PAG is antinociceptive,^19,34,42^ and this effect is known to be mediated via the CB1 cannabinoid receptor.^12^ The other cannabinoid receptor, CB2, is mainly expressed by peripheral immune cells.^49^ However, CB2 receptor is also expressed by neurons and glial cells within the brainstem and DH, and upon activation alleviates inflammatory pain hypersensitivity.^14,25^ Additionally, a nonclassic cannabinoid receptor, GPR55, is expressed by both neuronal and glial cells within the central nervous system (CNS).^45^ The study of GPR55-mediated pain modulation is still in its infancy,^24,52,54^ and roles in both pronociception and antinociception upon receptor activation were observed.^52^

It is widely accepted that apart from pain modulation, the EC signalling system serves a trophic role in utero, guiding neuronal and glial migration, axon elongation, and synaptogenesis.^27,51^ More importantly, the role of the EC signalling system in neurodevelopment and maturation continues after birth,^23,37^ as prolonged exposure to the psychoactive cannabinoid of marijuana, D-9-tetrahydrocannabinol during adolescence causes plastic changes in the hippocampus.^47^ Given the role of the EC signalling system in pain modulation and neurodevelopment, we sought to investigate the role of the EC system on postnatal maturation of pain processing.

Here, we report significant age-dependent plasticity within the supraspinal EC system that alters DH nociception. This article maps these changes for the first time in both rodent and human brains at a molecular and functional level. These data have profound implications for our understanding of supraspinal control of spinal nociception and the treatment of pain in early life.

## 2. Materials and methods

### 2.1. Animals

Postnatal day (P)3, 14, and 40, Sprague-Dawley rats were purchased from Charles River, United Kingdom. These ages were chosen for our study, as it spans across early to late postnatal maturation of the rat, and pain behaviours reach maturity around P40. Pups were housed with their dams in individually ventilated cages in an in-house animal facility. Free access to food and water was available throughout. All experiments were performed in P10, P21, and P40 rats during the animals' light cycle. Experimental procedures were performed under the Home Office License 40/3647 and in accordance with the Animals (Scientific Procedures) Act 1986 and IASP guidelines. Whilst we acknowledge that this term is controversial and that rats at this age/weight are not fully adult, they display many of the behavioural, physiological, and anatomical responses seen in adults and are routinely used as adults in the entire pain literature.

### 2.2. Surgery

Periaqueductal grey and RVM microinjection animals were anaesthetised with isoflurane (Baxter, Newbury, Berkshire, United Kingdom) and mounted on a stereotaxic frame (Kopf Instruments, Tujunga, CA). The skull was exposed and the bregma was located. Stereotaxic coordinates for the ventral PAG (vPAG) and RVM were calculated (PAG: both P40 and P21: left-right [L-R] 0.5 mm; anterior-posterior [A-P] −7.8 mm; dorsal-ventral [D-V] −6.0 mm; P10: L-R 0.5 mm; A-P −7.8 mm; DV −4.5 mm; L-R 0.5; A-P −7.8 mm; D-V −4.5 mm: RVM: P40: L-R 0 mm; D-V −10 mm; A-P −9.7 mm; P21: L-R 0 mm; D-V −10 mm; A-P −9.2 mm; and P10: L-R 0 mm; D-V −8 mm; A-P −8.7 mm), and a 26-gauge 2.5-μL syringe (Hamilton, Reno, NV) was inserted through a drilled hole in the skull. Drugs were injected over a 5-minute period, after which the syringe was removed and the wound was closed. Total volume of drug administered into the PAG was 1 and 0.5 μL to the RVM at P21 and P40 with 0.5 μL (PAG) and 0.25 μL (RVM) being administered to P10 rats in accordance with previously published studies^30,39^; only 1 drug was administered per animal. Total brain volume is the same between P21 and P40 (see Ref. 29), and therefore, the same volume for injections was used.

### 2.3. Electromyographic recordings

Anaesthesia (isoflurane) in P21 and P40 rats was maintained with a surgically implanted endotracheal cannula, whereas in P10 rats, it was maintained with a fitted nose cone. Isoflurane concentration was kept at 1.3% to maintain light anaesthesia, as previously described.^29,39^ The fur overlying the biceps femoris muscle was trimmed and a bipolar concentric needle electromyographic (EMG) recording electrode (comprising a modified 27-gauge hypodermic needle; Ainsworth, Coventry, United Kingdom) inserted into the belly of the muscle. The EMG electrode was connected to a NeuroLog head-stage (NL100AK; Digitimer, Welwyn Garden City, United Kingdom), signals amplified ×2000 (NL104A), band-pass filtered between 10 and 1000 Hz (NL125) before being sampled at 2 kHz using LabChart software via a PowerLab data acquisition unit (AD Instruments Ltd, Oxford, United Kingdom). In these experiments, spinal reflex excitability was determined by the EMG activity of flexor hind limb muscle evoked by mechanical stimulation of the plantar hind paw using von Frey hairs (vFh).

Responses to 2 subthreshold vFh (T − 1 and T − 2) and the threshold hair (T) and a suprathreshold hair (T + 1) were recorded and the same 4 hairs used in all subsequent stimulation conditions for data analysis. Thresholds were determined as the vFh that produced an EMG response more than 10% greater than the resting EMG activity. Each hair was then applied 3 times, and the mean reading for each of the 3 presentations recorded. Different hairs were used in each age group as mechanical withdrawal thresholds increase with age. A stimulus-response curve of EMG magnitude vs mechanical stimulus intensity was plotted, and the area under the curve was calculated to provide an integrated measure of spinal reflex excitability. Specific hairs used in each age were (P10: 15, 26, 60, and 100 g; P21: 26, 60, 100, and 180 g; and P40: 60, 100, 180, and 300 g).

### 2.4. Immunohistochemistry

P10, P21, and adult rats were overdosed with intraperitoneal (i.p.) injection of sodium pentobarbital (P21 and adults, 2 mL; P10, 1 mL). Animals were then transcardially perfused with 4% paraformaldehyde and brains quickly dissected. The PAG and the RVM were sectioned (40 μm) on a freezing microtome (Leica, SM2010R).

Tissue was blocked with 3% serum with 0.3% triton X100 (Sigma Aldrich, United Kingdom) for 1 hour before incubation with primary antibody. The primary antibodies used were goat anti-CB1 (Frontier Institute, Tokyo, Japan, 1:200^32^). This antibody has been validated previously in rat tissue. Sections were incubated with this antibody overnight at room temperature. After incubation with the primary antibody, sections were incubated with Alexa Fluor–conjugated (Invitrogen, Loughborough, United Kingdom) secondary antibodies (1:500) for 2 hours at room temperature.

Immunofluorescent sections were observed with a Leica IRE2 fluorescence microscope fitted with a Hamamatsu Orca-ER monochrome camera and captured using Volocity 6.1 software (Perkin Elmer, Llantrisant, United Kingdom). Same exposure time of image acquisition was used for each sections staining for the different antibodies from the different animals to ensure consistent brightness in images. Image J 1.29 (National Institutes of Health, Bethesda, MD) was used to adjust brightness and contrast of the images postacquisition. Systematic random sampling and unbiased stereological methods were used for quantification semiquantitative analysis as adopted from previously published studies.^26,41^

### 2.5. TaqMan real-time PCR

Human brain tissue was obtained from the Nottingham Biobank. Ethical approval for this study was sought and granted (Study ACP0000100), and studies were conducted in Human Tissue Act–registered (2004) laboratories. Tissue was selected to come from cases aged 25 to 29 weeks of gestation, 38 to 39 weeks of gestation, and older than 2 years. Cause of death was provided and included transcervical ascending infection/foetal inflammatory syndrome, uteroplacental perfusion deficiency (1 with concomitant *Streptococcus* B infection), osteoskeletal dysplasia, multiple congenital malformations, placental villous dysmaturity, or no significant abnormality. Tissue was provided as paraffin-embedded blocks, including the midbrain, pons, and medulla. Real-time (RT) PCR was conducted on 10 μm sections from each block following the same procedure as described below. Primers and probes for the human sequences were designed in an analogous manner to that stated below (in the paragraph after next).

P10, P21, and P40 rats were overdosed with i.p. injection of sodium pentobarbital (P21 and P40s, 2 mL; P10, 1 mL). Their brains were quickly dissected out on ice. Tissue from the PAG and RVM was isolated. Samples were flash frozen in liquid nitrogen and stored at −80°C.

TaqMan quantitative RT-PCR was performed using the StepOnePlus Real-Time PCR System (Applied Biosystems, Forster City, CA). Primers and probes for glyceraldehyde 3-phosphate dehydrogenase (GAPDH; National Center for Biotechnology Information [NCBI] reference sequence NM_017008.3), CB1 receptor (NM_012874.4), CB2 receptor (NC_005104.4), GPR55 receptor (XM_006245494.1), NAPE-PLD (NM_199381.1), and DAGLα (NM_006133.2) were designed on Primer Express 3 (Applied Biosystems). Each sample was run in triplicates. Expression of target genes was normalised to GAPDH, and expression of target genes was determined using the relative standard curve method. All sequences for primers were validated with a BLAST search.

### 2.6. Liquid chromatography–mass spectrometry analysis of endocannabinoids

P10, 21, and 40 rats were killed by overdose of i.p. sodium pentobarbital, and PAG and RVM tissue were dissected and frozen in liquid nitrogen.

A liquid chromatography–mass spectrometry method was used for analysis of ECs based on a previously published method [69]. Internal standards of 100 μL of 2-arachidonoylglycerol (2-AG)-d8 (10 μM) and 15 μL of AEA-d8 (28 μM) were added to each tissue sample or EC standards and vortexed briefly. Ethyl acetate: hexane (9: 1 vol/vol) was added to each sample, vortex mixed (10 minutes), and centrifuged (13,000 rpm, 10 minutes, 4°C). The procedure was repeated and the supernatants pooled and evaporated using a vacuum centrifugal evaporator. Prior to analysis, each sample extract was reconstituted in 100 μL of acetonitrile. The injection volume was 10 μL. EC standards (AEA, 2-AG, oleoylethanolamine [OEA], and palmitoylethanolamine [PEA] and internal standards 2-AG-d8 and AEA-d8) were purchased from Axxora Laboratory services, Bingham, Nottingham, United Kingdom. The HPLC system used was a Shimadzu SCL-10Avp (Shimadzu, Columbia, MD) coupled to a triple quadrupole ion-trap 4000 QTRAP mass spectrometer (AB SCIEX, Warrington, United Kingdom) equipped with Turbo Spray ionisation interface. Analytes were separated chromatographically on a Waters Symmetry C18 column (internal diameter 100 × 2.1 mm, particle size 3.5 μm) with a mobile phase flow rate of 0.3 mL/min. Multiple-reaction monitoring of individual compounds using specific precursor and product mass-to-charge (*m/z*) ratios allowed simultaneous measurement of AEA, 2-AG, OEA, and PEA. Quantification was by the internal standard method with extracted calibration standards, and data analysis was performed using Analyst v 1.4.2 (AB SCIEX).

### 2.7. Statistics

All individual data points were represented as mean ± SEM. Electromyographic data were normally distributed. Statistical comparisons between the age groups and drugs were made using 2-way analysis of variance (ANOVA) or 1-way ANOVA with Bonferroni multiple comparisons. Statistical comparisons between the age groups for the expression of various endocannabinoid targets in mass spectrometry, TaqMan, and RT-PCR experiments were made using 1-way ANOVA with Bonferroni multiple comparisons.

## 3. Results

### 3.1. Expression of ECs is developmentally regulated in the periaqueductal grey and rostroventral medulla

**Figure 1.**
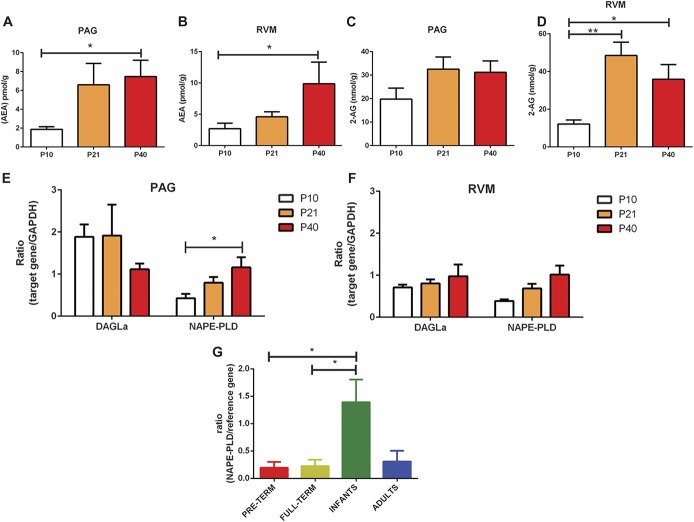
Changes in the expression of endocannabinoids (eCBs) and eCB-synthesising enzymes in the brainstem nuclei of the rat and human midbrain during postnatal development. (A and B) Mass spectrometry analysis of anandamide levels in the periaqueductal grey (PAG) and rostroventral medulla (RVM). Expression of anandamide increased during early postnatal development and reached maturity by postnatal day (P)21. (*P* < 0.05 in the PAG and RVM; 1-way analysis of variance [ANOVA].) (C and D) Mass spectrometry analysis of 2-arachidonoylglycerol (2-AG) levels in the PAG and RVM. No changes in the expression of 2-AG were observed in the PAG (C). Expression of 2-AG increased during early postnatal development and reached maturity by P21 in the RVM (D; *P* < 0.01; 1-way ANOVA). (E) Taqman real-time (RT) PCR analysis of the eCB-synthesising enzymes NAPE-PLD and DAGLα in the ventral PAG. Expression of NAPE-PLD mRNA increased during early postnatal development and reached maturity by P21 (*P* < 0.05; 1-way ANOVA). (F) Taqman RT-PCR analysis of NAPE-PLD and DAGLα in the RVM. No significant changes were observed. (G) Expression of NAPE-PLD mRNA was highest in the human infant midbrain compared with all other age groups tested (*P* < 0.01; 1-way ANOVA). Nine to 11 animals per age group used for mass spectrometry experiments. Three to 4 animals per age group for Taqman RT-PCR and immunohistochemical experiments. Four to 8 human midbrain tissue per age group used for TaqMan RT-PCR. Data shown here represent mean ± SEM. * and ** = *P* < 0.05 and *P* < 0.01, respectively, between age comparisons, 1-way ANOVA with Bonferroni multiple comparisons.

We first measured the concentrations of endogenous CB1 receptor ligands, anandamide (AEA) and 2-AG, in the PAG and RVM of postnatal day (P)10, P21, and P40 rats by tandem liquid chromatography–mass spectrometry. In the PAG (Fig. 1A) and RVM (Fig. 1B), the levels of AEA significantly increased during the early postnatal period and reached mature levels by P21. Similarly, 2-AG levels in the PAG (Fig. 1C) and RVM (Fig. 1D) also increased and reached mature levels by P21. Consistent with previous reports, the measured concentration of 2-AG is higher compared with anandamide in the brain regions tested, as AEA is measured in pmol/g range, whereas 2-AG in nmol/g range.^8^ Collectively, these data indicate that the expression of ECs increases with postnatal age of the rat.

Oleoylethanolamine and PEA were also measured (supplemental Fig. 1, available online at http://links.lww.com/PAIN/A471). Although OEA and PEA lack affinity at CB1 and CB2 receptors,^10^ they exert similar effects as other synthetic cannabinoids, via the peroxisome proliferator–activated receptor-alpha, GPR55, and GPR119 receptors.^12,55^ There were no changes in PEA levels in the PAG (supplemental Fig. 1A, available online at http://links.lww.com/PAIN/A471), but PEA levels in the RVM increased with postnatal age and were highest in P40s (supplemental Fig. 1B, available online at http://links.lww.com/PAIN/A471). The concentration of OEA in the PAG and RVM also increased with postnatal age, and similarly, expression was highest in P40s (supplemental Fig. 1C, D, available online at http://links.lww.com/PAIN/A471).

We next assessed whether the expression of endocannabinoid-synthesising enzymes, N-acyl phosphatidylethanolamine–specific phospholipase D (NAPE-PLD; synthesising anandamide) and diacylglycerol lipase alpha (DAGLα; synthesising 2-AG), also increases with postnatal age. In the PAG and RVM, we undertook PCR investigations. We isolated mRNA from the ventrolateral PAG (vPAG) (Fig. 1E) and the RVM (Fig. 1F) of P10, 21, and P40 rats. We focused on the vPAG as neurons from this region indirectly project to the DH via the RVM.^3,59^ The transcript levels of target genes were normalised to the expression of GAPDH in the same tissue, and quantification was performed using the relative standard curve method.^39^

In the vPAG, NAPE-PLD mRNA transcript levels increased as the rats aged, and NAPE-PLD mRNA transcript levels were higher in P40s compared with P10 (Fig. 1E). No differences in DAGLα mRNA transcript levels (Fig. 1E) were observed in the vPAG between the ages. In the RVM, no differences in DAGLα and NAPE-PLD mRNA transcript levels were observed between the ages (Fig. 1F).

To investigate whether our results are translational in humans, we also measured mRNA levels of NAPE-PLD in the midbrain of postmortem human tissue deposited in the Nottingham Biobank by TaqMan qRT-PCR. Tissues were selected to span a range of ages from subjects who were born preterm (24-26 weeks postconceptional age, n = 8), full term (39-40 weeks postconceptional age, n = 8), infants (3-8 months, n = 8), and adults (14-60 years, n = 4). mRNA levels were measured using a geometric mean of reference genes (cyclophilin A and hydroxymethylbilane synthase). We found that NAPE-PLD mRNA transcript level was higher in infants than in both preterm and full term (Fig. 1G), which suggests that like the rat, expression of EC-synthesising enzymes undergoes significant postnatal modulation during development in humans.

### 3.2. Expression of cannabinoid receptors also undergoes significant postnatal refinement

Results from our previous experiments implied that the expression of ECs within the brainstem nuclei is upregulated as rats mature. Therefore, we sought to understand whether there is also postnatal modification of EC receptors within these brainstem nuclei. Expression of the CB1, CB2, and GPR55 receptors in the rat was examined using TaqMan RT-PCR. The expression of CB1 and CB2 receptors in the human midbrain was studied using TaqMan RT-PCR and in situ hybridisation techniques.

**Figure 2.**
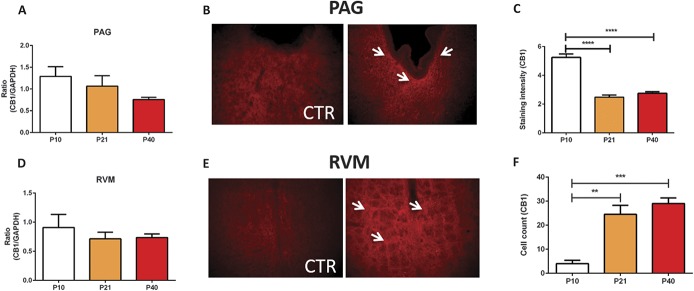
Changes in the expression of endocannabinoid receptors CB1, CB2, and GPR55 in the brainstem of the rat during postnatal maturation. (A) TaqMan real-time (RT) PCR analysis of CB1 receptor mRNA in the ventral periaqueductal grey (vPAG) of P10, P21, and P40 rats, no statistically significant changes were observed between the ages. (B) Fluorescent image (×20 magnification) and quantification (C) of CB1 immunoreactivity in the vPAG. White arrows denote CB1-specific terminal staining. The expression of CB1 receptors in the vPAG decreased as the animals aged, as immunoreactivity was highest at P10 (*P* < 0.0001; 1-way analysis of variance [ANOVA]). TaqMan RT-PCR analysis of CB1 receptors in the rostroventral medulla (RVM) of P10, P21, and P40 rats, no changes were observed between the ages. (E) Fluorescent image (×20 magnification) and quantification (F) of CB1 immunoreactivity in the RVM. White arrows denote CB1-specific terminal staining. The expression of CB1 receptors in the RVM increased as the animals aged (*P* < 0.01-0.001; 1-way ANOVA) (C and D) N = 3 to 5 animals per age group. CTR, control. Data shown here represent mean ± SEM. **, ***, and **** = *P* < 0.01, *P* < 0.001, and *P* < 0.0001, respectively, between age comparisons, 1-way ANOVA with Bonferroni multiple comparisons.

**Figure 3.**
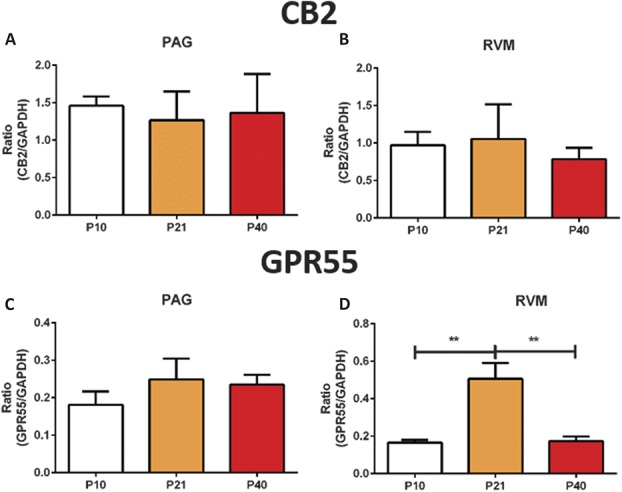
No changes in CB2 transcript levels in the periaqueductal grey (PAG) and rostroventral medulla (RVM) whilst the expression of the GPR55 gene is developmentally regulated. (A and B) TaqMan RT-PCR analysis oCB2 receptor mRNA in the PAG and RVM, respectively, no significant changes in were detected. (C and D) TaqMan RT-PCR analysis of GPR55 receptor mRNA in the PAG and the RVM of P10, P21, and P40 rats (*P* < 0.001; 1 way analysis of variance [ANOVA]). No changes were observed in the PAG, but GPR55 mRNA was transiently upregulated at P21 in the RVM (D). N = 3 to 5 animals per age group. Data shown here represent mean ± SEM. ** = *P* < 0.01, between age comparisons, 1-way ANOVA with Bonferroni multiple comparisons.

In the rat PAG, CB1 mRNA transcript levels decreased with age, but this trend did not reach statistical significance (Fig. 2A). Immunohistochemistry demonstrated that CB1 staining was mostly diffuse fibres (Fig. 2B), found at the axonal terminals, consistent with previously published findings.^60^ CB1 immunoreactivity was particularly dense in the region immediately adjacent to the cerebral aqueduct. Staining intensity analysis showed that CB1 receptor immunoreactivity decreased as the rats and was highest at P10 (Fig. 2C). In the rat RVM, no age-related differences in CB1 mRNA transcript levels were observed (Fig. 2D). However, using immunohistochemistry, we found significant differences in the expression pattern of CB1 within the nucleus raphe magnus, specifically during postnatal development. Immunoreactivity for CB1 receptors increased during postnatal development and reached adult levels by P21 (Figs. 2E and F). No differences in CB2 receptor mRNA transcript levels were observed in the vPAG between the ages (Fig. 3A). Similarly, we found no changes in CB2 transcript levels in the RVM (Fig. 3B). No changes in the GPR55 mRNA expression were observed in the PAG (Fig. 3C), but significant postnatal refinement to the expression of GPR55 mRNA was detected in the RVM with levels transiently and significantly upregulated at P21 (Fig. 3D).

**Figure 4.**
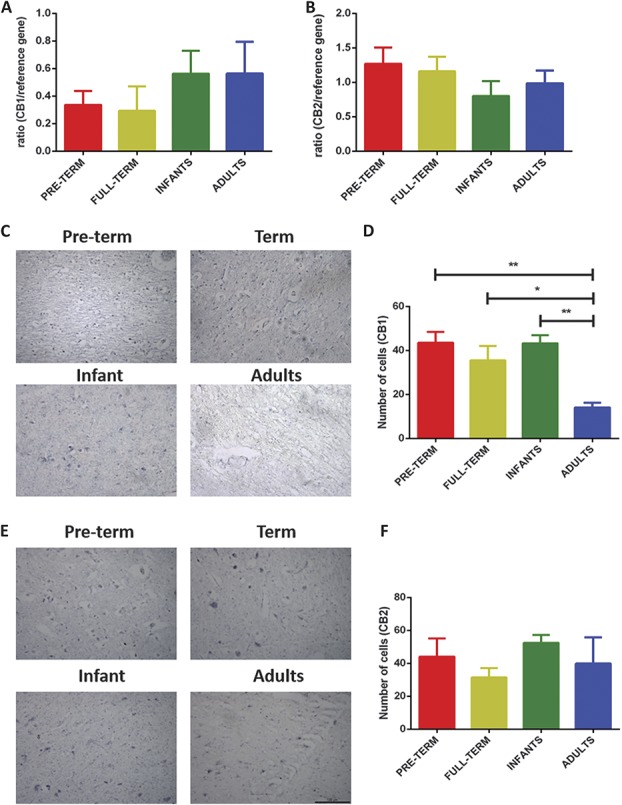
Changes in the expression of endocannabinoid receptors CB1 and CB2 in the human midbrain during postnatal maturation. (A and B) TaqMan real-time (RT) PCR analysis of CB1 and CB2 receptor mRNA, no significant changes in were detected. (C) In situ hybridisation images (×20 magnification) of CB1 receptors in preterm, term, infant, and P40 midbrain. (D) Quantification by cell-counting analysis showed that the expression of CB1 receptors is higher during early postnatal development compared with P40 (*P* < 0.01; 1-way analysis of variance [ANOVA]). (E) In situ hybridisation images of CB2 receptors. (F) No significant changes in the expression of CB2 receptors were observed throughout postnatal development. Four to 8 human midbrain tissue per age group was used for TaqMan RT-PCR and in situ hybridisation, respectively. Data shown here represent mean ± SEM. * and ** = *P* < 0.05 and *P* < 0.01, respectively, between age comparison, 1-way ANOVA with Bonferroni multiple comparisons.

In the human midbrain, no changes in the expression of either CB1 or CB2 receptor mRNA were observed (Figs. 4A and B). To examine the pattern of mRNA expression in human tissue, in situ hybridisation techniques were used to investigate the expression of CB1 and CB2 receptors in the midbrain of human preterm, full term, infants, and P40s (Figs. 4C–F). Counting of CB1-positive cells revealed that the expression of CB1 receptors is lowest in P40s compared with earlier ages (Figs. 4C and D). There were no differences in the CB1 receptor mRNA expression between preterm, full term, and infants. There were also no changes in the expression of CB2 receptor protein between the age groups tested (Figs. 4E and F).

These findings suggest that unlike the ECs and EC-synthesising enzymes, the expression of CB1, CB2, and GPR55 receptors within the PAG is not developmentally regulated; however, within the RVM, GPR55 mRNA transcription is increased for a short period around P21. Our results also show that in the human midbrain, the expression of CB1 receptors undergoes significant postnatal refinement and is highest during early infancy and childhood.

### 3.3. CB1/CB2 receptors in the brainstem nuclei inhibit nociceptive reflex in both P21 and P40 rodents

The functional significance of the developing EC signalling system on nociceptive reflexes was investigated by pharmacologically activating cannabinoid receptors within the brainstem nuclei in lightly anaesthetised rats at different developmental time points. Various synthetic cannabinoids were microinjected into either the vPAG or the RVM of each rat, and EMG recordings were performed before and after drug administration in the bicep femoris muscle to assess withdrawal reflexes to mechanical stimulation of the hind paw using calibrated vFh. In our first experiment, WIN55212 (4 μg, CB1 and CB2 receptor agonists) was microinjected into the PAG of postnatal day (P)21 and P40 rats, as previously experiments showed a functional switch in descending pain modulation between these ages (supplemental Fig. 2, available online at http://links.lww.com/PAIN/A471).^29,30,39,53^ Intra-PAG WIN55212 significantly reduced spinal reflex excitability (supplemental Fig. 2C, available online at http://links.lww.com/PAIN/A471) and increased mechanical withdrawal thresholds (supplemental Fig. 2D, available online at http://links.lww.com/PAIN/A471) in both P21 and P40 rats compared with vehicle controls. The increase in mechanical withdrawal threshold was significantly greater in P40 rats than P21.

**Figure 5.**
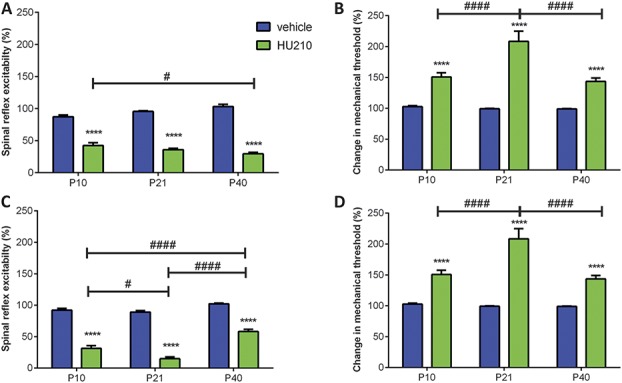
Effect of intraperiaqueductal grey (PAG) and rostroventral medulla (RVM) microinjection of HU210 (CB1/2 receptor agonist, 4 μg/animal) on withdrawal reflexes to mechanical von Frey hair stimulation in P10, P21, and P40 rats. (A) Intra-PAG HU210 significantly decreased spinal reflex excitability compared with vehicle responses in all ages tested (*P* < 0.0001; 2-way analysis of variance [ANOVA]). This effect was more pronounced in P10 animals compared with P40s. (B) Intra-PAG HU210 significantly increased mechanical thresholds in P21 and P40 rats (*P* < 0.0001; 2-way ANOVA). This effect is more pronounced in P21 and P40 animals compared with P10. (C) Intra-RVM HU210 decreased spinal reflex excitabilities compared with vehicle responses in all ages tested (*P* < 0.0001, 2-way ANOVA). This effect was strongest in P21 animals. (D) Intra-RVM HU210 significantly increased mechanical thresholds compared with vehicle responses in all ages tested (*P* < 0.0001, 2-way ANOVA). This effect was strongest for P21 animals. Four to 8 animals per drug per age group. Data shown here represent the mean ± SEM. **** = *P* < 0.0001, between drug comparisons, 2-way ANOVA with Bonferroni multiple comparisons. # and #### = *P* < 0.05 and *P* < 0.0001, respectively, between age comparison, 2-way ANOVA with Bonferroni multiple comparisons.

To further investigate the consequences of activating cannabinoid receptors on nociceptive reflexes, HU210 (4 μg, CB1 and CB2 receptor agonists) was microinjected injected into the vPAG or the RVM of P10, P21, and P40 rats (Fig. 5). Periaqueductal grey microinjection of HU210 potently reduced spinal reflex excitability compared with vehicle controls in all age groups tested (Fig. 5A). A reduction in spinal reflex excitability was accompanied by an increase in mechanical withdrawal threshold (Fig. 5B). The same pattern was seen when HU210 was microinjected in the RVM (Figs. 5C and D). Intra-PAG HU210-induced antinociceptive effects were stronger in P21 and P40 animals compared with P10 (Figs. 5A and B), with the greatest effect of HU210 being observed at P21 (Figs. 5C and D and supplemental Figure 3; http://links.lww.com/PAIN/A471).

Collectively, these data showed that functional activation of CB1 and CB2 receptors within the descending pain pathway is antinociceptive throughout postnatal development. In addition, some age-related differences were observed (increased efficacy of HU210 at P21), which suggests that EC signalling via the CB1 and CB2 receptors undergoes significant refinement over the early postnatal period.

### 3.4. Supraspinal GPR55 receptor–mediated analgesia in early life

**Figure 6.**
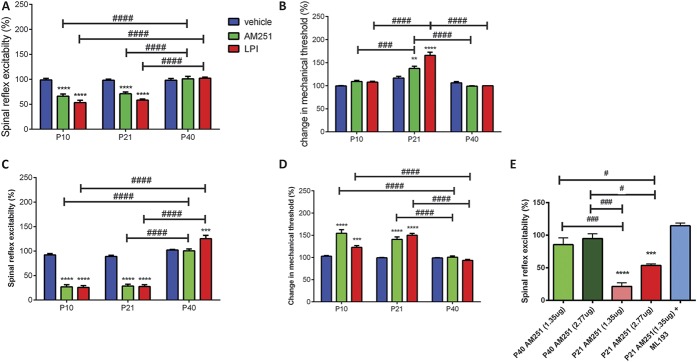
Effect of intraperiaqueductal grey (PAG) and rostroventral medulla (RVM) microinjection of AM251 (CB1 receptor inverse agonist, GPR55 receptor agonist, 2.77 and 1.35 μg/animal) and lysophosphatidylinositol (LPI) (endogenous GPR55 agonist, 12 μg/animal) on withdrawal reflexes to mechanical von Frey hair stimulation in P10, P21, and P40 rats. (A) Intra-PAG AM251 and LPI only decreased spinal reflex excitability in P10 and P21 rats (*P* < 0.0001, 2-way analysis of variance [ANOVA]). There are significant age-related differences in AM251 and LPI responses between P40 and younger animals (*P* < 0.0001, 2-way ANOVA). (B) Intra-PAG AM251 and LPI did not change the mechanical thresholds in either P10 or P40 rats, but significantly increased it in P21 animals. There are significantly age-related differences in AM251 and LPI-mediated changes in mechanical withdrawal thresholds (*P* < 0.001, 2-way ANOVA). (C) Intra-RVM AM251 did not have an effect in P40 rats, but significantly reduced spinal reflex excitabilities in P10 and P21 animals. Lysophosphatidylinositol increased spinal reflex excitability in P40 rats, but decreased it in P10 and P21 animals. These age-related differences were significant (*P* < 0.0001, 2-way ANOVA). (D) Intra-RVM AM251 and LPI significantly increased mechanical thresholds in P10 and P21 rats, but had no effect in P40 animals. This age-related difference was significant (*P* < 0.0001, 2-way ANOVA). (E) Intra-PAG AM251 (2.77 and 1.35 μg) did not have an effect in P40, but significantly inhibited spinal reflex excitability in P21 animals (*P* < 0.0001, 1-way ANOVA). Coadministration of AM251 (1.35 μg) with the GPR55 receptor–specific antagonist ML193 (1 μg) reversed AM251-mediated inhibition of spinal reflex excitability in P21 animals. **, ***, and **** = *P* < 0.01, *P* < 0.001, and *P* < 0.0001, respectively, between drug comparisons, 2-way ANOVA and 1-way ANOVA with Bonferroni multiple comparisons. #, ### and #### = *P* < 0.05, *P* < 0.001 and *P* < 0.0001, respectively, between age comparison, 2-way ANOVA and 1-way ANOVA with Bonferroni multiple comparisons.

We next tested the effects of decreasing cannabinoid receptor activity on nociceptive processing during postnatal maturation. Intra-PAG microinjection of AM251 (2.77 and 1.35 μg, CB1 receptor inverse agonist) had no effect on spinal reflex excitability in P40 rats compared with vehicle controls (Fig. 6A) and had no effect on mechanical withdrawal threshold. However, intra-PAG AM251 (2.77 μg) significantly inhibited spinal reflex excitability in P10 and P21 rats. Mechanical withdrawal thresholds were increased after intra-PAG AM251 in P21 rats only (Fig. 6B). Similarly, intra-RVM AM251 (2.77 μg) significantly reduced spinal reflex excitability and increased mechanical withdrawal thresholds in P10 and P21 rats compared with vehicle controls (Figs. 6C and D). This result is surprising, as both agonist (HU210) and inverse agonist (AM251) mediate the same effect on spinal nociception in younger animals.

In addition to a role as a CB1 receptor inverse agonist, AM251 is also known to activate GPR55 receptors.^33^ Lysophosphatidylinositol (LPI) is known to be an endogenous agonist of this receptor.^44^ Similar to the effect of AM251, intra-PAG microinjection of LPI (12 μg) significantly reduced spinal reflex excitability in P10 and P21 rats compared with vehicle controls (Fig. 6A) and significantly increased mechanical withdrawal thresholds in P21 rats (Fig. 6B). Intra-RVM LPI significantly reduced reflex excitability in P10 and P21 rats compared with controls (Fig. 6C) and increased mechanical withdrawal thresholds in both P10 and P21 rats (Fig. 6D). In P40 rats, neither intra-PAG nor intra-RVM microinjection of LPI had an effect on mechanical withdrawal thresholds, but intra-RVM LPI significantly increased spinal reflex excitability when compared with vehicle controls.

To fully elucidate whether AM251 was acting via the GPR55 receptors and address the concern of using a higher dose (2.77 μg), we assessed the effects of low-dose (1.35 μg) AM251, on spinal excitability in P21 and P40 rats when administered to the vlPAG. Similar to our previous observations, low-dose AM251 (1.35 μg) had no effect in the P40 but reduced spinal reflex excitability in P21 rats (Fig. 6E). Next, we tested the effect of blocking GPR55 receptors in the vlPAG of P21 rats with the GPR55 antagonist, ML193 (1 μg). Coadministration of ML193 with AM251 (1.35 μg) reversed the ability of AM251 to decrease spinal excitability in P21 rats (Fig. 6E). Moreover, a significant decrease in spinal reflex excitability was observed with both the lower (1.35 μg) and higher doses (2.77 μg) compared with P21 animals receiving coadministration of AM251 and ML193. These findings provided further support in the role of GPR55 on antinociception via descending control systems early in life.

## 4. Discussion

Previous research has identified several postnatal modifications within the pain pathway that affect on the development of functional nociception. These include alterations in terminations of primary afferent sensory neurons within the DH,^6^ immature neuroimmune interactions,^5^ and a fundamental shift in supraspinal control over DH excitability, from predominantly excitatory in early life to inhibition after the third postnatal week.^29,53^

As described elsewhere, the EC signalling system is essential for normal brain maturation and pain processing.^36^ In our studies, we report postnatal alternations in the expression of EC-related targets in the midbrain of humans and the brainstem nuclei of rats, and these changes have significant functional consequences on pain modulation. Interestingly, our data also reveal a novel role for GPR55 receptors in pain modulation during the early postnatal period, as the expression of GPR55 receptor mRNA was highest in the early adolescent period of the rat, and supraspinal activation of GPR55 receptors strongly inhibited nociceptive reflexes in early infancy and adolescence of the rat. Altogether, our research provides further insights into our understanding in the maturation of pain signalling systems during postnatal development.

### 4.1. Expression of endocannabinoids within the brainstem nuclei during postnatal development

This is the first study of its kind to measure both the expression of EC and EC targets within the brainstem nuclei in both rat and human brains. Our major finding is that ECs in the rat PAG and RVM increases with postnatal age. Liquid chromatography–mass spectrometry analysis showed significant increases in the levels of AEA in both the PAG and the RVM; in parallel with this, the expression of NAPE-PLD mRNA in the PAG increased as the animals aged and reached maturity by the third postnatal week in the rat. In humans, the expression of NAPE-PLD mRNA in the midbrain was highest in infants compared with both preterm and full-term neonates. Altogether, these findings suggest that the expression of AEA increases within the brainstem nuclei of mammals during postnatal development. Importantly, because of the nature of human tissue we obtained for this study, early life inflammation could also contribute to changes in the expression NAPE-PLD mRNA transcript levels during infancy. Therefore, future experiments with a different cohort of human tissue would provide further insight.

The levels of other ECs, such as 2-AG, also increase with postnatal age in the RVM of the rat. In our study, only subtle changes were detected in the expression of the 2-AG–synthesising enzyme, DAGLα. No changes were detected in the expression of DAGLα mRNA within the brainstem nuclei. The disparity between DAGLα expression pattern and levels of 2-AG may be explained by the greater availability of the substrate for the production of 2-AG or to age-related posttranslational modifications; it has been shown that 2-AG can be synthesised independently of DAGLα activity: PIP_2_ can be catalysed into 2-arachidonoyl-lysophospholipid by phospholipase A1, which is in turn hydrolysed by lyso-PLC to become 2-AG.^46^

### 4.2. Expression of cannabinoid receptors within the brainstem nuclei during postnatal development

We did not find significant changes in the expression of either CB1 or CB2 receptor mRNA with postnatal age, in both rats and humans. However, in situ hybridisation experiments showed that the number of CB1 receptor–positive cells in the human midbrain was highest during infancy, and immunohistochemical expression of CB1 receptor underwent postnatal modification within the brainstem nuclei of the rat. In line with previous published findings,^7,17^ within the rat PAG, CB1 receptor expression was found closely along the lining of the aqueduct, and staining intensity decreased as the animals aged. We also found an increase in CB1 receptor immunoreactivity in the RVM, The disparity between mRNA and immunohistochemical data implies that changes in expression and function of the receptor may reflect differences in the translation of mRNA rather than changes in gene transcription. Additionally, it should be recognised that immunohistochemically verified visualisation of receptor may, in cases of fibre staining, reflect alterations in expression within cells in other CNS centres that innervate the PAG or RVM. Nonetheless, the expression of CB1 receptors at the protein level decreased in the human midbrain and rat PAG with postnatal age.

GPR55 mRNA was detected in all the regions tested (Fig. 6). The overall expression was lower compared with other endocannabinoid-related targets. Within the RVM, GPR55 mRNA peaked at P21, which again echoes the theme that P21 is a critical time point when developmentally regulated changes occur. In this study, immunohistochemical localisation of GPR55 receptors was attempted but because of technical reasons was unsuccessful. Validation of the quantification of the mRNA levels of CB2 and GPR55 receptors was attempted with immunohistochemistry as part of this wide-ranging study. However, it was impossible for us to validate the specificity of the antibodies against their respective targets, and therefore, these data are not presented in this article.

### 4.3. Functional role of cannabinoid receptors in nociception during postnatal development

In this study, we examined the role of cannabinoid receptors in the brainstem on nociceptive processing during postnatal maturation. We pharmacologically manipulated cannabinoid receptor activity by in vivo intracerebral microinjections. Whilst we specifically targeted the vlPAG and RVM, there is a slight possibility that the drugs we administered could potentially diffuse into neighbouring regions within the brainstem. Nonetheless, we showed that activation of CB1/2 receptors in the brainstem regions at any of the ages tested was always inhibitory. Initially, WIN55212 was injected into the PAG of P21 and P40 rats, which was antinociceptive in both ages. HU210 was used in subsequent experiments as a CB1/CB2 agonist because HU210 is approximately a 100-fold more potent at the CB1 receptors than WIN55212 (Ki for CB1 receptors, HU210 = 0.061 nM, WIN55212 = 62.3 nM). Application of HU210 in the PAG and the RVM also reduced nociceptive behaviours in all ages tested, further indicating that CB1/CB2 agonism is analgesic throughout postnatal development.

These findings imply that although the expression of components of the ECs changes with postnatal age, EC-dependent nociceptive processing within the descending pathway via CB1 and CB2 receptors is not developmentally regulated. This is in line with observations from previous studies, where systematic administration of CB1/CB2 agonists, including WIN55212, CP55940, and HU210 inhibited nociceptive responses in young and adult rats.^9,19,35,50^ More importantly, it is known that the opioid and cannabinoid signalling system work synergistically within pain modulatory pathways.^11,13,61^ Given that (1) significant postnatal refinement occurs within opioid-mediated pain modulation pathway,^30,39^ and (2) ECs play a trophic role in development, it is likely that the increase in ECs contributes to maturation of opioid signalling during the postnatal period, and this possibility warrants further investigation.

We also demonstrate an exciting new cannabinoid target for the treatment of pain in early life. Changes in the physiological functions of EC signalling system were revealed when we activate GPR55 receptors, by microinjecting LPI into the PAG and the RVM. Notably, intra-PAG or RVM administration of LPI reduced spinal reflex excitability and increased mechanical withdrawal thresholds in P10 and P21 rats, whereas in P40s, a mild increase in spinal reflex excitability was observed after intra-RVM microinjection of LPI. The effect of LPI on P40 spinal reflex excitability reported in our study is line with observations in other studies. We further substantiated the age-dependent role of GPR55-mediated signalling in the PAG by blocking the ability of AM251 to produce decreased spinal excitability in P21 rats with the specific GPR55 receptor antagonist ML193. The general consensus is that GPR55 receptor–mediated activity is most likely to be pronociceptive in mature animals.^15^ The receptor in the mature CNS is coupled to the Gq proteins and when activated causes an increase in intracellular calcium levels and synaptic excitability,^15,57^ whereas CB1 activation negatively couples to adenylate cyclase activity via Gi proteins. It has also been shown that intraplantar injection of LPI (2 pmol) leads to allodynia in P40 mice.^24^

Nonetheless, results from this study indicated that LPI in P40 RVM was pronociceptive, whereas that in the immature PAG and RVM was antinociceptive, which suggests a switch in GPR55-mediated actions throughout postnatal development. Recently, it has been shown that GPR55 expressed on primary afferent nociceptors is activated by lyso-phosphatidyl-β-D-glucoside, released from spinal radial glia.^27^ This interaction was essential for the maturation of spinal nociceptive circuits, as disruption of it led to misallocation of nociceptive axons into proprioceptive zones in the DH. Supraspinal GPR55 receptors may also have a similar neurodevelopmental role, and this warrants further investigation.

### 4.4. Role of the EC signalling system in maturation of pain processing during the postnatal period

In summary, our data imply that the EC signalling system is a promising target for pain management in immature patients. Activation of CB1 and CB2 receptors in the brainstem nuclei is always antinociceptive, regardless of postnatal age. In addition, activation of supraspinal GPR55 receptors is only antinociceptive in young animals, revealing fundamental development in the physiological functions of the EC signalling system during postnatal development, and GPR55 receptors as a novel target for paediatric analgesic.

Concurrent with the postnatal development of the physiological functions of the EC signalling system, anatomical studies imply that the expression of ECs within the brainstem nuclei increases with postnatal age. This anatomical refinement may be important for endogenous pain inhibition throughout the postnatal period, and mediating the maturation of synaptic connections within the pain signalling pathway.

## Conflict of interest statement

The authors have no conflict of interest to declare.

This work was supported by the Biotechnology and Biological Sciences Research Council [grant number BB/I001565/1], and by a BBSRC PhD studentship.

## Supplementary Material

SUPPLEMENTARY MATERIAL

## Appendix A. Supplemental digital content

Supplemental digital content associated with this article can be found online at http://links.lww.com/PAIN/A471.
