# Supplementary figures and images for: Age-dependent plasticity in endocannabinoid modulation of pain processing through postnatal development
Source: Pain. 2017 Aug 1;158(11):2222–32. doi: 10.1097/j.pain.0000000000001027 (PMC5642337; doi:10.1097/j.pain.0000000000001027)

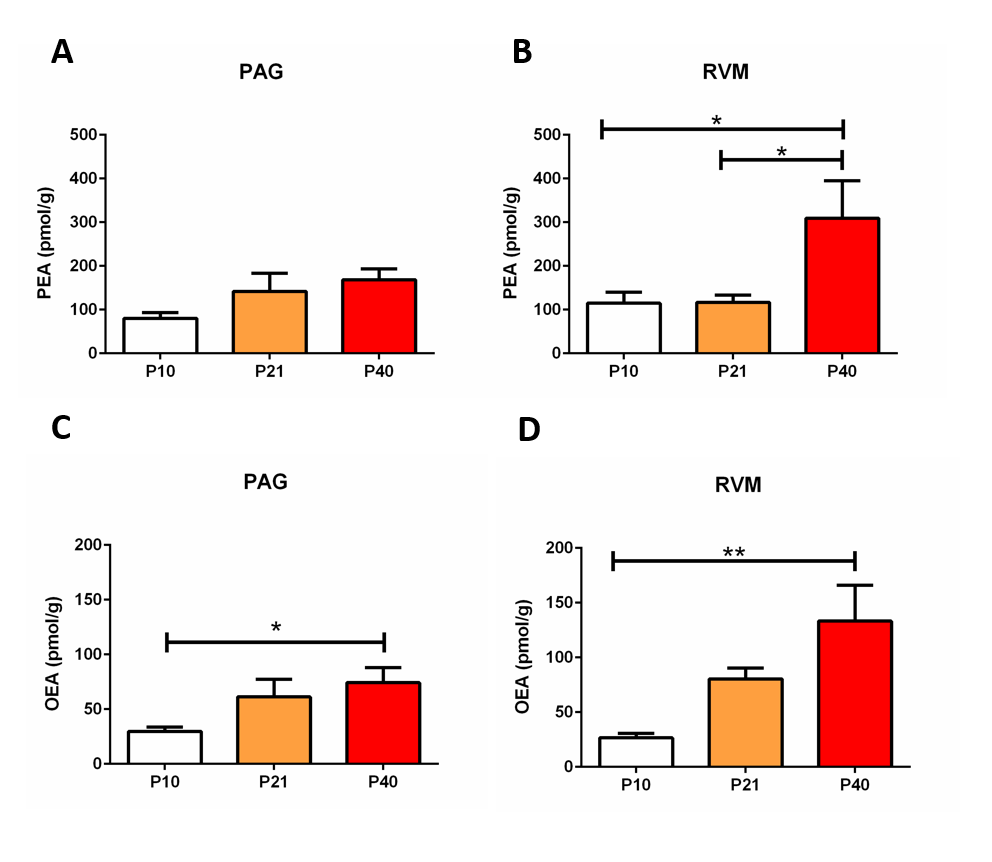

Supplement: SUPPLEMENTARY MATERIAL [file jop-158-2222-s001.tif]

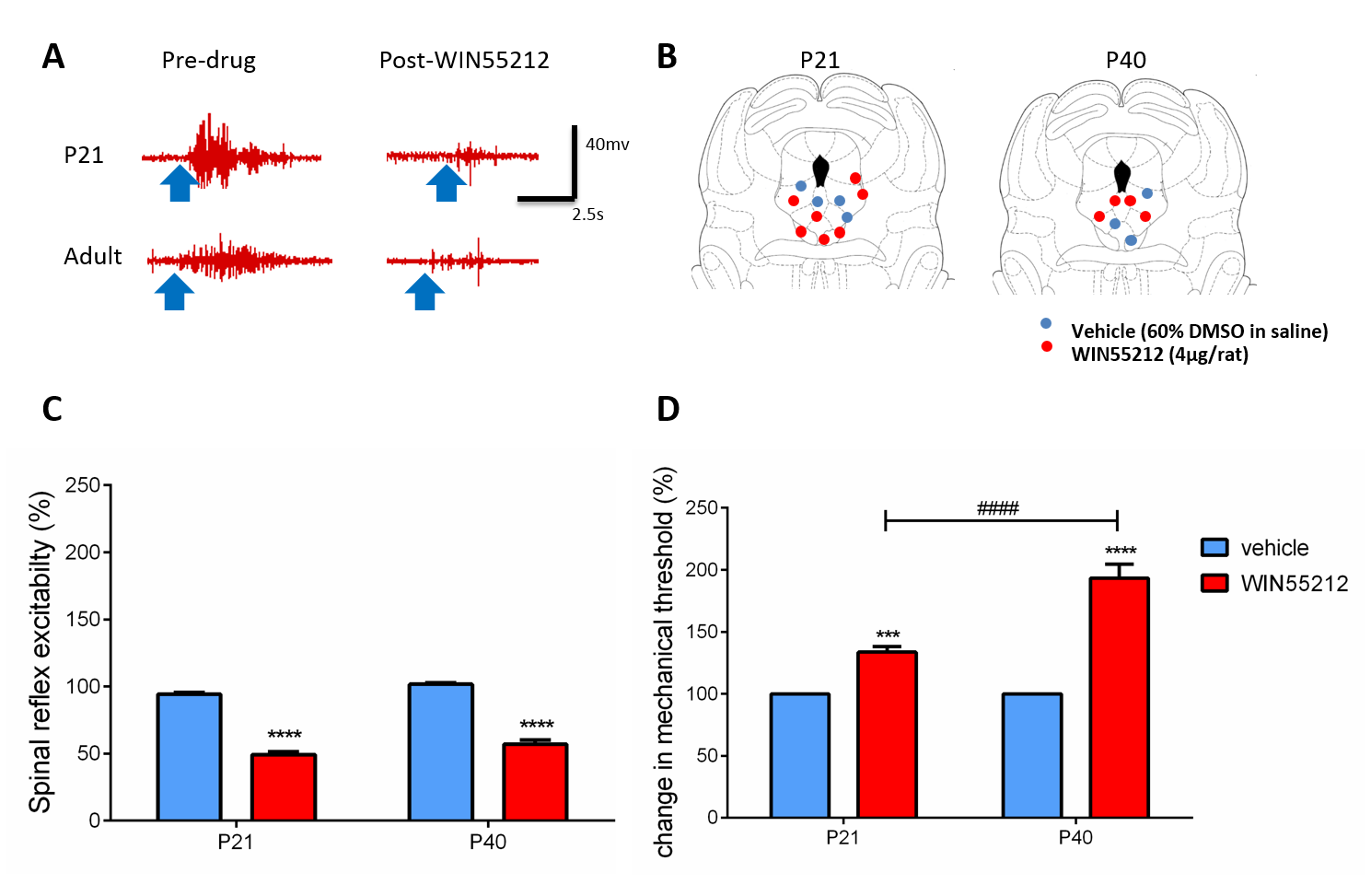

Supplement: SUPPLEMENTARY MATERIAL [file jop-158-2222-s002.tif]

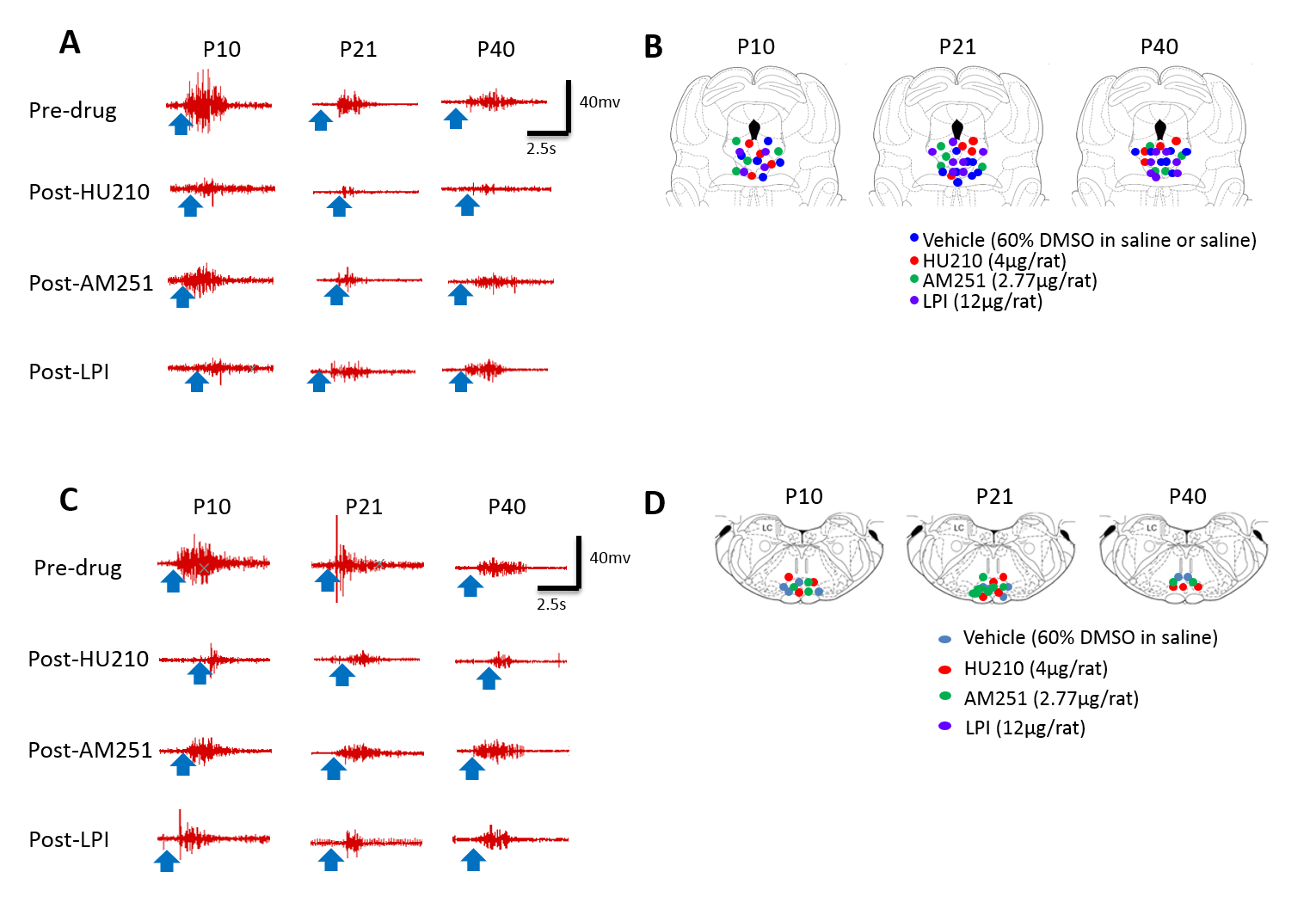

Supplement: SUPPLEMENTARY MATERIAL [file jop-158-2222-s003.tif]
